# Enhancing LGMD-based model for collision prediction via binocular structure

**DOI:** 10.3389/fnins.2023.1247227

**Published:** 2023-09-05

**Authors:** Yi Zheng, Yusi Wang, Guangrong Wu, Haiyang Li, Jigen Peng

**Affiliations:** ^1^School of Mathematics and Information Science, Guangzhou University, Guangzhou, China; ^2^Machine Life and Intelligence Research Center, Guangzhou University, Guangzhou, China

**Keywords:** collision prediction, lobula giant movement detectors (LGMDs), binocular vision, disparity, depth distance

## Abstract

**Introduction:**

Lobular giant motion detector (LGMD) neurons, renowned for their distinctive response to looming stimuli, inspire the development of visual neural network models for collision prediction. However, the existing LGMD-based models could not yet incorporate the invaluable feature of depth distance and still suffer from the following two primary drawbacks. Firstly, they struggle to effectively distinguish the three fundamental motion patterns of approaching, receding, and translating, in contrast to the natural abilities of LGMD neurons. Secondly, due to their reliance on a general determination process employing an activation function and fixed threshold for output, these models exhibit dramatic fluctuations in prediction effectiveness across different scenarios.

**Methods:**

To address these issues, we propose a novel LGMD-based model with a binocular structure (Bi-LGMD). The depth distance of the moving object is extracted by calculating the binocular disparity facilitating a clear differentiation of the motion patterns, after obtaining the moving object's contour through the basic components of the LGMD network. In addition, we introduce a self-adaptive warning depth-distance, enhancing the model's robustness in various motion scenarios.

**Results:**

The effectiveness of the proposed model is verified using computer-simulated and real-world videos.

**Discussion:**

Furthermore, the experimental results demonstrate that the proposed model is robust to contrast and noise.

## 1. Introduction

In the real world, collisions often lead to some kind of danger and unexpected loss. Therefore, many modern artificial machines, such as ground vehicles and unmanned aerial vehicles (UAVs), should be equipped with the intellectual abilities of collision prediction. Current methods for collision prediction, such as laser, infrared, radar, and ultrasonic, are not very suitable for daily civilian machines because of the disadvantages of high price, large size, high power consumption, and so on. Meanwhile, vision-based sensors, with the characteristics of economy and energy saving, have gradually become one of the most mainstream methods of sensing collision in the past decades. However, in terms of effectiveness and robustness, it still needs to be further improved (Mukhtar et al., 2015).

As we know, in nature, many insects show excellent collision prediction and collision avoidance abilities based on visual information, which benefits from their millions of years of evolution (Eichler et al., 2017). Despite their minuscule and simple brains, these lowly creatures seem to hold the key to solving some of mankind's greatest problems (Franceschini, 2014; Xu et al., 2023), and bring us some inspirations to build a collision prediction neural network based on visual information (Serres and Ruffier, 2017; Fu et al., 2018a). Among these insects, locusts are the most representative. When locust plague breaks out, millions of locusts can travel hundreds of miles together free of collision (Kennedy, 1951). Researchers observe that when a collision is imminent, locusts can respond quickly and change their flight direction in a very short time (hundreds of milliseconds; Fu et al., 2019c). How do locusts achieve it?

Lobula giant movement detector (LGMD), which is a huge single neuron located on the third visual neuropile of the lobule, was found by O'Shea and Williams (1974). LGMD neuron responds vigorously to approaching objects while producing little or no response to receding ones (O'shea and Rowell, 1976; Sztarker and Rind, 2014; Wernitznig et al., 2015; Rind et al., 2016). Further, researchers conduct a lot of experiments and explorations around the reflection properties of LGMD neuron (Gabbiani and Krapp, 2006; Dewell and Gabbiani, 2018, 2019; Zhu et al., 2018), and the results show that the LGMD neuron is an ideal model for constructing collision prediction visual neural network.

Based on these biological experiments, Rind and Bramwell (1996) proposed an LGMD-based neural network model. The model is composed of four groups of cells—photoreceptor cells (*P* cells), excitatory cells (*E* cells), inhibitory cells (*I* cells), and summing cells (*S* cells), as well as two single cells—feed-forward inhibition and LGMD. Since then, Yue and Rind (2006) introduced an extra artificial layer (*G* layer) to extract the extended edge of the approaching object by enhancing the cluster output, which improved the model's performance and achieved ideal results in real-world scenarios.

Following the above two studies, a large number of LGMD-based visual neural network models have sprung up. For example, based on ON/OFF channels (Fu, 2023), Fu et al. (2019b) realized the special selectivity to darker looming objects in brighter background in the model, which simulated the response of LGMD2 neurons in the infancy of locusts. Inspired by the visual pathway of Drosophila, Li et al. (2022) added a contrast channel to the LGMD-based model, which improved the stability of the model under different contrasts. Luan et al. (2021, 2022) used a similar network model to build a visual neural network with the ability to encode spatial position information, and successfully simulated MLG1 neurons in crabs. Zhao et al. (2018, 2019, 2021) further optimized the original model by designing the temporal and spatial distribution in the model according to the latest discovery of locust anatomical synaptic connection, which was successfully applied to UAV agile flight. Some models are also be tested in ground vehicle scenarios (Hartbauer, 2017; Fu et al., 2019a), mobile robots (Hu et al., 2016; Čížek et al., 2017), and recently in UAVs (Poiesi and Cavallaro, 2016; Salt et al., 2017, 2019) and micro robots (Fu et al., 2020, 2021). In addition, it is also embodied in hardware implementation, such as the FPGA (Meng et al., 2010).

However, the current models lack the consideration of the depth distance of moving objects, which is certainly a highly valuable feature for collision prediction tasks. This absence of depth distance information in the existing models results in several shortcomings. First, existing models are not able to distinguish well between the three fundamental motion modes of approaching, receding and translating, resulting in their inability to consistently demonstrate a preference for approaching objects. Secondly, the response result of the models is heavily influenced by activation function parameters and corresponding given hard thresholds. Thirdly, the models are sensitive to various input image stream factors, including noise and contrast. While some models enhance certain aspects by designing artificial mechanisms, extracting the core feature of depth distance holds the potential to effectively address all of these issues simultaneously.

For that, a novel LGMD-based neural network model with binocular vision is proposed in this paper, named Bi-LGMD. This model requires two image stream inputs, coming from the left and right eye, respectively. For both inputs, a basic LGMD-based model is used to extract the contours of the moving object. Then, based on the principle of binocular stereo vision, the obtained contour information is used to compute the disparity of the moving object, and the moving object's depth distance at each time step is further estimated. Based on this, motion patterns can be effectively distinguished. Moreover, different from existing models, the activation function is not required in our model. Instead, the concept of warning depth-distance is introduced. Depending on the change of the estimated depth distance at each time, the warning depth-distance is dynamically and adaptively adjusted through a specific computational rule. The LGMD neuron is activated only when an approaching object reaches the warning depth-distance. Hence, the parameter setting problem for the activation function is avoided. More importantly, the model is more robust to input image streams. On the one hand, this is due to the consideration of more essential kinematic features of depth-distance. On the other hand, the computational process of disparity is mainly based on the matching of two contours from the left and right channel, rather than the pixel value itself, so the factors that seriously affect the pixel value of an image (such as noise, contrast, etc.) have a great impact on existing models, but the computational result of disparity is relatively stable.

The main innovations of this paper can be summarized as follows:

1. This paper proposes a novel LGMD-based model with binocular structure, and the essential feature of depth distance is introduced into the model for the first time. As a result, the proposed model is able to clearly distinguish motion modes such as approaching, receding and translating, with improved selectivity.
2. We design a dynamic adaptive warning depth distance related to the approaching velocity. On the one hand, the model could be adapted to more complex approaching modes. On the other hand, the model does not rely on the activation function parameters and a given hard threshold, alleviating the extreme sensitivity of the existing models to activation parameters.
3. Unlike existing models that heavily rely on the pixel values of G layer outputs, the proposed model ultimately focuses on matching the overall left and right outputs. Based on this novel perspective, the proposed model has stronger robustness to factors such as noise and contrast in the input image streams.

The rest of this paper is organized as follows. Section 2 introduces some related work, including motion pattern recognition in the model and the advantages of incorporating stereo vision. Section 3 describes the proposed Bi-LGMD visual neural network. Systematic experiments and analyses of the model results are illustrated in Section 4. Thereafter, further discussions are given in Section 5. Section 6 concludes the paper.

## 2. Related work

### 2.1. Motion pattern recognition

LGMD neuron is viewed as an ideal paradigm for constructing collision prediction models. Numerous LGMD-based models are validated to indeed respond significantly to looming stimuli, yet it is difficult to be completely unresponsive to other motion patterns. Therefore, further improvements are still needed to clearly distinguish between the basic motion patterns including approaching, receding and translating.

In the past, some models attempted further improvements in terms of the selective response of the model to motion patterns, for example, Lei et al. (2022) improved the LGMD-based model using the ON-OFF competition mechanism, enabling it to distinguish a looming object from a near and fast translatory moving object. However, it does not explore the response to receding stimuli, and the competition mechanism does not seem to be effective in distinguishing between approaching and receding. Fu et al. (2018b) designed a spike frequency adaptation (SFA) mechanism to enhance the collision selectivity to approaching objects, however, the model still has a brief and small response to the receding and translating stimuli, which may cause false alarms in situations where the model parameters are inappropriate (especially the activation parameter and spiking threshold).

In general, while some models could make partial discrimination between different motion patterns, there are still some problems, such as how to choose the spiking threshold. By contrast, the trend of depth distance is the most intuitive way to distinguish basic motion patterns. Once it is effectively estimated, the model can understand the motion patterns more “visually,” knowing exactly which of the “approaching, receding, and translating” the motion pattern belongs to at the current moment. The results of the discrimination of motion modes will no longer be affected by parameters and thresholds, and its discrimination method is obviously simple, robust, and interpretable.

### 2.2. Binocular structure and stereo vision

Binocular vision, which allows for depth perception, is crucial for arthropods to interact with their environment. This is particularly important for behaviors such as motion navigation, prey capture, and attack avoidance (Nityananda et al., 2016a; Scarano et al., 2018). The binocular structure of arthropods is capable of processing information from both eyes to estimate depth and distance in the visual scene through a concept known as “disparity” (Parker, 2007; Nityananda et al., 2016b). Recent research on arthropods, like crabs, has shown a strong binocular coupling between their eyes indicating the use of binocular depth vision in capturing prey (Horridge and Sandeman, 1964; Scarano et al., 2018). Praying mantises, for example, use their stereoscopic vision to estimate the distance to their prey. Once it is within reach, they trigger a rapid strike of their forelegs (Rossel, 1986; Rosner et al., 2019).

Although the computational mechanisms behind binocular vision in arthropods are not yet fully understood, experimental findings indicate that different types of neurons in the Lobula region of their brains compute binocular information (Rosner et al., 2020). Rosner and colleagues have provided evidence that individual neurons in the praying mantis brain can recognize specific binocular information such as disparity and eccentricity, allowing them to determine locations in three-dimensional space. They identified the existence of disparity-sensitive neurons in the insect's brain and proved their role in the development of stereo vision (Rosner et al., 2019).

Interestingly, stereoscopic vision in insects, including mantises, differs from that of humans. Insects rely on changes in luminance rather than luminance directly to perceive depth (Rosner et al., 2019), which implies that insects pay more attention to moving and changing visual information rather than static details in the background. This unique approach allows insects like praying mantises to develop an efficient stereoscopic vision system using a visual network of neurons that is significantly smaller than the human brain (Rossel, 1983; Collett, 1996).

Therefore, the introduction of binocular structures in LGMD-based neural networks to extract depth-distance information is intuitively significant for enhancing collision prediction. Indeed, there has been some research work related to binocular LGMD modeling. For example, Yue and Rind (2009) proposed a network model with two LGMD modules for near-range path navigation. In their work, the input image will be decomposed into left and right parts for the two LGMD modules, and the two outputs will be compared in terms of strength and weakness to determine which way the robot's wheels should dodge. In addition, Fu et al. (2017) also designed similar binocular structures using LGMD1 and LGMD2 to investigate how this combined strategy performs for different visual stimuli when applied to a robot. However, it appears that there are few models based on LGMD that utilize binocular structures to develop stereo vision, extract depth-distance information, and explore the advantages of incorporating such information into LGMD-based models.

## 3. Formulation of the model

In this section, the proposed model and the corresponding computational methods are described in detail. Here, we first introduce the overall framework of the Bi-LGMD model, and then give a more specific description in the following sections.

**Figure 1:**
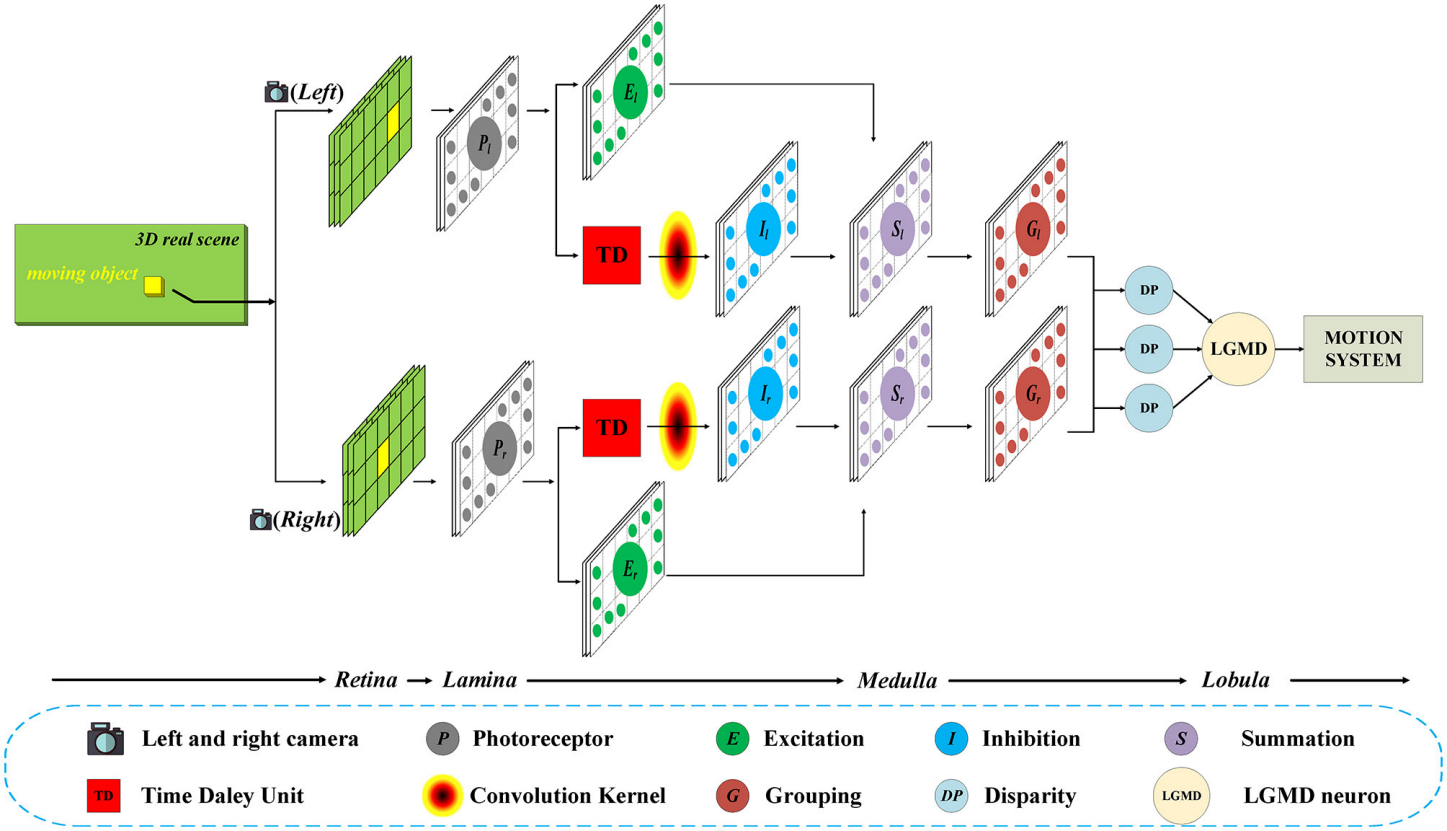
Schematic of the Bi-LGMD visual neural network. For visual stimulation in the three-dimensional world, the left and right cameras are used to shoot at the same time, and the two input image streams are processed separately in the early stage. In the last Medulla to Lobula layer, all the information is integrated through the disparity principle, so as to extract the depth distance information of the moving object. Finally, the LGMD neuron responds based on the changes in estimated depth distance.

As shown in Figure 1, in general, the proposed model contains two parallel channels to process the input image stream from the left and right camera, respectively. Each channel consists of five layers, including photoreceptor (*P*), excitation (*E*), inhibition (*I*), summation (*S*), and grouping (*G*) layers. Then, the outputs of the two parallel channels will be integrated in the disparity (*DP*) layer, and the information will eventually be transmitted to the LGMD layer.

In this model, inputs from both cameras are considered equally important. Therefore, the two parallel channels have exactly the same structure and the same calculation method, and the relevant parameters are set to be the same in the subsequent experiments. For convenience, in the following sections, the subscripts *l* and *r* are used to represent that the corresponding variables belong to the left and the right channel, respectively. In the following basic process, we describe the computational method in the left channel as an example, which is exactly the same as in the right channel.

### 3.1. Basic process

The basic process includes *P*, *E*, *I*, *S*, *G* layers. This classical process framework has been used in many existing models, such as Fu et al. (2019b, 2020), Luan et al. (2021), Lei et al. (2022), and Wang et al. (2023). In fact, our model does not change significantly for this part, so we will briefly review it here.

#### 3.1.1. P layer

In this layer, the photoreceptors are arranged as a matrix. Each photoreceptor captures the grayscale luminance of the corresponding pixel in the input image stream and computes the temporal difference between the sequence frames to preliminarily extract motion information. The mathematical formula can be defined as


(1) $$\begin{array}{l} P_{l}\left(x,y,t\right)=L_{l}\left(x,y,t\right)-L_{l}\left(x,y,t-1\right)+\overset{n_{p}}{\underset{i=1}{\sum }}a_{i}P_{l}\left(x,y,t-i\right) \end{array}$$

where *L*(*x, y, t*) stands for the grayscale luminance of the pixel (*x, y*) at time *t*, and *P*(*x, y, t*) represents the grayscale luminance change; *n*_*p*_ indicates the maximum number of frames the persistence of the luminance change could last, and *a*_*i*_ is a decay coefficient, which is defined by


(2) $$\begin{array}{l} a_{i}={\left(1+e^{i}\right)}^{-1} \end{array}$$

#### 3.1.2. IE layer

The *IE* layer is the core of the “critical race” mentioned by Rind and Bramwell (1996). Both excitatory cells (*E* cells) and lateral inhibitory cells (*I* cells) receive the outputs of the *P* cells. *E* cells directly receive the excitation from the corresponding *P* cells without temporal latency, while the *I* cells, which pass inhibition, receive the excitation from the surrounding adjacent *P* cells by convolving, and there is one image frame time-delay. The mathematical formulas are defined as follows:


(3) $$\begin{array}{l} E_{l}\left(x,y,t\right)=P_{l}\left(x,y,t\right) \end{array}$$

(4) $$\begin{array}{l} I_{l}\left(x,y,t\right)=\overset{1}{\underset{i=-1}{\sum }}\overset{1}{\underset{j=-1}{\sum }}P_{l}\left(x+i,y+j,t-1\right)w_{I}\left(i,j\right) \end{array}$$

where *E*(*x, y, t*) and *I*(*x, y, t*) are the activity of excitatory cells and lateral inhibitory cells, respectively. *w*_*I*_ is the local inhibition weight that meets the following matrix, which is also used in Yue and Rind (2006), Fu et al. (2018b), Luan et al. (2021), and Li et al. (2022).


$$w_{I}=\left(\begin{array}{ccc} 0.125 & 0.25 & 0.125\\ 0.25 & 0 & 0.25\\ 0.125 & 0.25 & 0.125 \end{array}\right)$$

#### 3.1.3. S layer

In the *S* layer, the information processing results of *E* cells and *I* cells in the upper layer need to be summarized. Here, a simple linear operation is adopted (Note that inhibition has the opposite sign against excitation):


(5) $$\begin{array}{l} S_{l}\left(x,y,t\right)=|E_{l}\left(x,y,t\right)|-|I_{l}\left(x,y,t\right)|*W_{I} \end{array}$$

where *W*_*I*_ is a constant which means global inhibition weight. In addition, since inhibition can reduce the activity of excitatory cells to 0 at most, it needs to be corrected here.


(6) $$\begin{array}{l} S_{l}\left(x,y,t\right)={\left[S_{l}\left(x,y,t\right)\right]}^{+} \end{array}$$

where [*x*]^+^ = *max*(0, *x*).

#### 3.1.4. G layer

**Figure 2:**
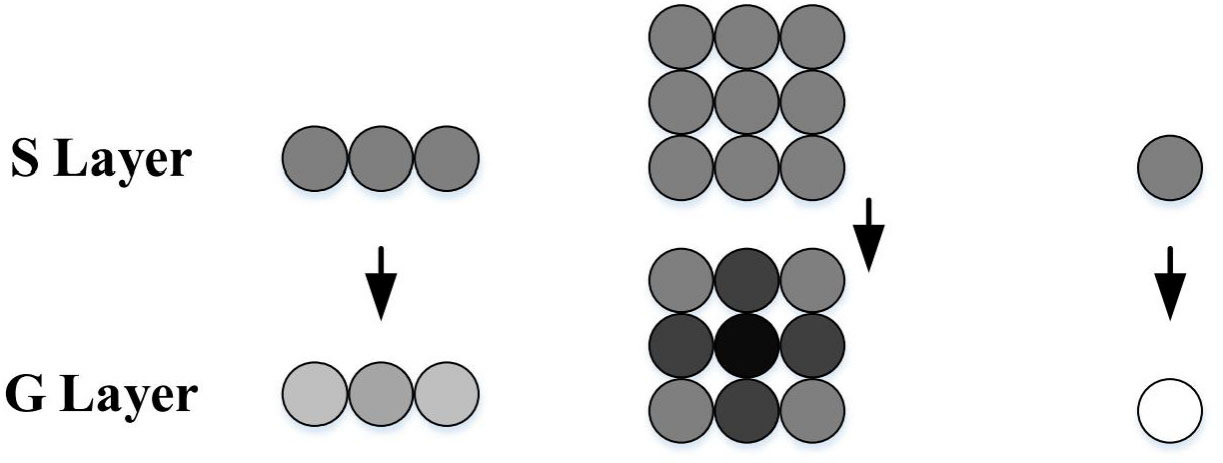
Schematic illustration of *G* layer processing, adapted from Yue and Rind (2006). The *S* cells surrounded by strong excitations obtain bigger passing coefficients, while the isolated ones gain smaller passing coefficients and may be ruled out by the threshold. The excitation strength is represented by gray levels, where the darker the color, the stronger the excitation.

To further enhance the outputs of the *S* layer, the *G* layer obtains a passing coefficient *Ce* through the cell's surrounding neighbors to filter out the isolated and decayed excitations, as illustrated in Figure 2. The computational formulas are as follows:


(7) $$\begin{array}{l} Ce_{l}\left(x,y,t\right)=\overset{1}{\underset{i=-1}{\sum }}\overset{1}{\underset{j=-1}{\sum }}S_{l}\left(x+i,y+j,t\right)w_{e}\left(i,j\right) \end{array}$$

(8) $$\begin{array}{l} G_{l}\left(x,y,t\right)=S_{l}\left(x,y,t\right)\cdot Ce_{l}\left(x,y,t\right)\cdot w_{l}{\left(t\right)}^{-1} \end{array}$$

(9) $$\begin{array}{l} w_{e}=\frac{1}{9}\times \left(\begin{array}{ccc} 1 & 1 & 1\\ 1 & 1 & 1\\ 1 & 1 & 1 \end{array}\right) \end{array}$$

(10) $$\begin{array}{l} w_{l}\left(t\right)=max\left({\left[Ce_{l}\right]}_{t}\right)\cdot C_{w}^{-1}+\Delta_{c} \end{array}$$

where *w* is a scale parameter computed at every time step. *C*_*w*_ is a constant. *max*([*Ce*]_*t*_) stands for the largest element in matrix [*Ce*]_*t*_. Δ_*c*_ is a small real number, which prevents the denominator from being 0 during calculation. Finally, a threshold *T*_*de*_ is introduced for the final calculation as follows.


(11) $$\hat{G_{l}}(x,y,t)=\{\begin{array}{ll} G_{l}(x,y,t), & if\;G_{l}(x,y,t)\geq T_{de}\\ 0, & otherwise \end{array}$$

Therefore, after the processing of the *G* layer, the grouped excitations in the *S* layer representing expanding edges become stronger, while the isolated excitations caused by background details are largely filtered out.

### 3.2. Disparity layer (DP layer)

It is well-known that many creatures in nature have two eyes. The binocular structure can produce stereo vision, and obtain the information of depth distance through the disparity, which can not be achieved by a single eye (Ayache, 1991; Yang et al., 2017; Vienne et al., 2018). In this section, we use this principle to estimate the depth distance of moving objects at each time step. For this purpose, the information from the left and right cameras will be integrated into the *DP* layer.

#### 3.2.1. Computing method of disparity

**Figure 3:**
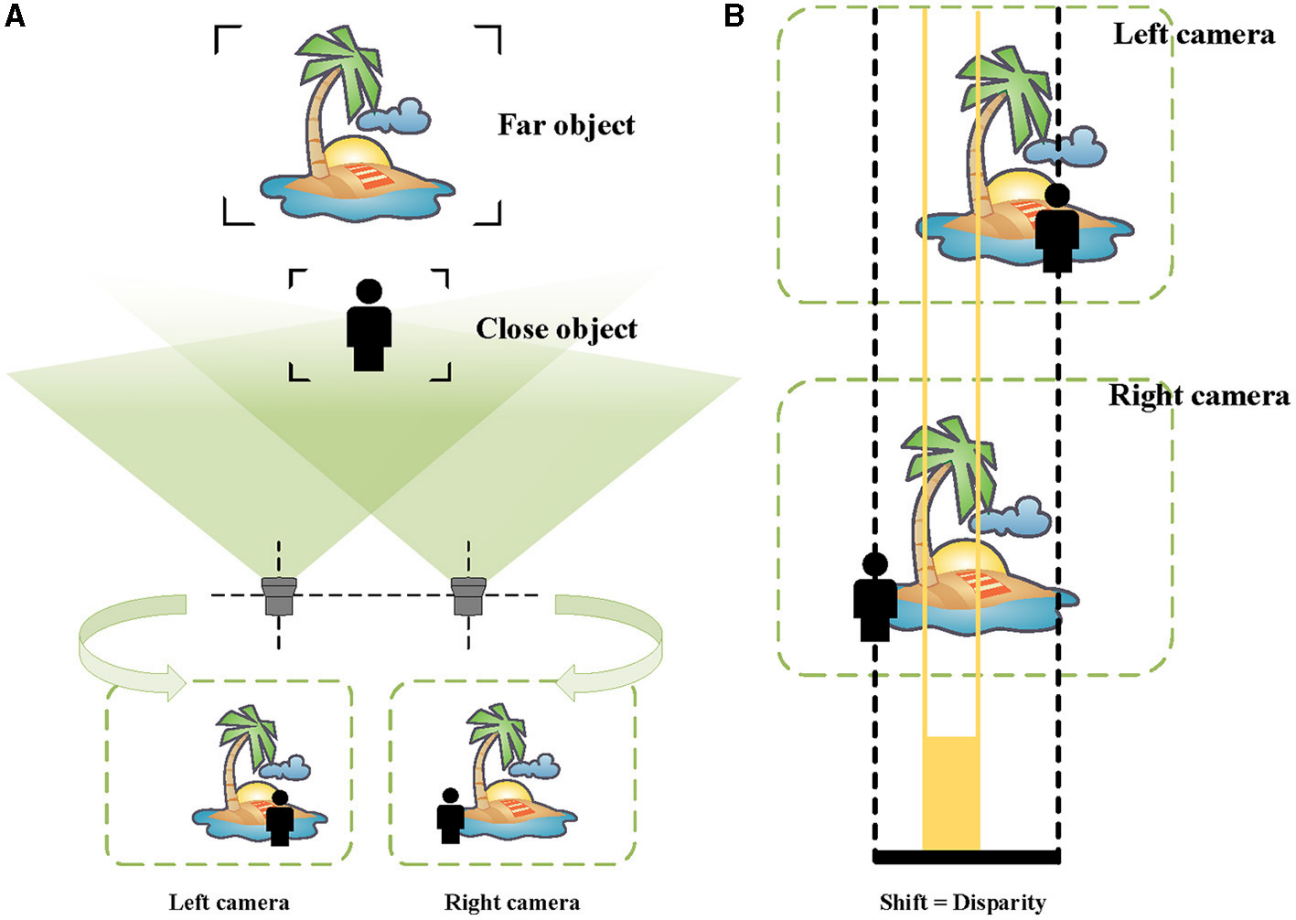
Schematic of binocular vision and disparity. **(A)** The images taken by the left and the right cameras. **(B)** For the far scenery, there is a small disparity, indicated by orange. For the person near, there is a large disparity, represented by black.

In the pictures taken by the left camera and the right camera, the imaging positions of the same object are different (see Figure 3A). More specifically, the imaging positions of closer objects are shifted considerably, while the difference is smaller for more distant objects. As shown in Figure 3B, this visual difference is called “disparity” (Ayache, 1991; Ding et al., 2021).

However, how to calculate the disparity in our model? Since the *G* layer mainly extracts the edge of the moving object, $\hat{G_{l}}$ and $\hat{G_{r}}$ can be used to obtain the disparity of the moving object. In the sense of the disparity described above, it can be computed by the following mathematical formula:


(12) $$\begin{array}{l} DP\left(t\right)=\underset{d}{\arg\max}\overset{R}{\underset{x=1}{\sum }}\overset{C-d}{\underset{y=1}{\sum }}\hat{G_{l}}\left(x,y+d,t\right)\cdot \hat{G_{r}}\left(x,y,t\right) \end{array}$$

where *DP* represents the pixel-level disparity, *R* and *C* denote the rows and columns of the input image size. Note that the formulation here follows the conventions used in the matrix so that the disparity is on the component *y*.

In theory, the search range of disparity *d* should be the entire image width. However, in practice, we can reduce the computational cost of the search process based on some clear facts. For example, since a moving object is always continuously changing in depth distance, the results at the previous time steps can be used as a reference and searched within a reasonable range. In addition, mathematically, this optimization function usually gets a larger calculation result near its optimal disparity, so we can also quickly find the optimal disparity by jumping search.

#### 3.2.2. Computing method of depth distance

Based on basic geometric knowledge, the depth distance between the object and the stereo cameras in the world coordinate system can be calculated using disparity. Specifically, the following relation holds when the stereo cameras with the same focal length are on the same horizontal line and the optical axes are parallel (Zhen et al., 2017; Sun et al., 2019):


(13) $$\begin{array}{l} D\left(t\right)=\frac{b\cdot f}{DP\left(t\right)\cdot pixelsize} \end{array}$$

where *D* stands for the depth distance of the object. *b*, *f*, *pixelsize* are constants, which can be obtained from the information of stereo cameras, representing the baseline length, focal length, and physical size corresponding to one pixel, respectively.

### 3.3. LGMD layer

After the DP layer, the proposed Bi-LGMD model is able to acquire the depth distance information. By comparing *D*(*t*) and *D*(*t*−1), the motion mode of the moving object at the current time *t* can be clearly distinguished (approaching, receding, and translating). However, to achieve a reasonable early warning response to the approaching movement, it is necessary to further judge whether the current approaching state is sufficiently dangerous. To this end, the early warning depth distance, an adaptive dynamic threshold, is introduced into our model.

#### 3.3.1. Warning depth distance (*D*_*W*_)

In fact, the proposed Bi-LGMD model also potentially extracts the approaching velocity information at each time step after *DP* layer. It is evident that faster moving objects require a greater warning depth-distance to ensure safety. Thus, the warning depth-distance should possess the following properties:


(14) $$\begin{array}{l} D_{W}\left(t\right)=F\left(D\left(t-1\right)-D\left(t\right)\right) \end{array}$$

where *F*(·) is a strictly monotonically increasing function.

There are many functions satisfying the above basic properties. For simply, linear functions are selected for discussion in this paper. Therefore, the specific formula is as follows:


(15) $$\begin{array}{l} D_{W}\left(t\right)=C_{T}\cdot \left(D\left(t-1\right)-D\left(t\right)\right) \end{array}$$

Although the linear function appears relatively simple, its implications are significant. The coefficient *C*_*T*_ holds a realistic physical interpretation, as it represents the time required for the machinery to avoid collisions, dependent upon individual machine attributes, such as flexibility in avoidance behavior. Consequently, if the moving object continues to approach at its current speed, the system will sound an early warning at the depth distance of *D*_*W*_, leaving the machine *C*_*T*_ time to avoid collisions. It is important to note that *D*_*W*_ is dynamically adaptive, adjusting the warning depth-distance accordingly in response to changes in approaching speed.

By the way, as a parameter with realistic physical meaning, *C*_*T*_ will be set within an appropriate range. If *C*_*T*_ is set too large, the system may trigger an alarm prematurely. On the other hand, if *C*_*T*_ is set too small, the machine may not have sufficient time to complete the avoidance maneuver.

#### 3.3.2. Activation of the LGMD neuron

In contrast to existing models that use the sigmoid function to produce activation values ranging from 0.5 to 1, our model employs a binary output: 0 and 1, representing the deactivation and activation of the LGMD neuron, respectively.

Specifically, the output of the LGMD layer is determined by two parts: one is whether the moving object is approaching, and the other is whether the moving object reaches the warning depth-distance *D*_*W*_. The output of the LGMD layer is 1 only if the above two parts are both true, and 0 otherwise. In this computational mode, only approaching objects are likely to activate the LGMD neuron, while objects in other motion modes are certainly not expected to activate it. Further, even if the object is in the process of approaching, the LGMD neuron will not be activated when the object does not reach the warning depth-distance. In other words, the approaching object is in a distant position and does not pose a collision threat for the time being, so the LGMD neuron does not need to be activated.


(16) $$LGMD(t)=\{\begin{array}{ll} 1, & if\;D(t)<D_{W}(t)\;and\;D(t)<D(t-1)\\ 0, & otherwise \end{array}$$

## 4. Experimental results and analysis

In this section, a series of systematic experiments will be performed from different aspects as comprehensively as possible. Also, some reasons for the experimental results will be analyzed in detail. All experiments can be divided into the following three categories: (1) Basic Synthetic Stimuli Testing, (2) Real Physical Stimuli Testing, and (3) Model Performance Testing. The state-of-the-art model (Fu et al., 2018b) will be used for comparison.

### 4.1. Experimental setup

**Figure 4:**
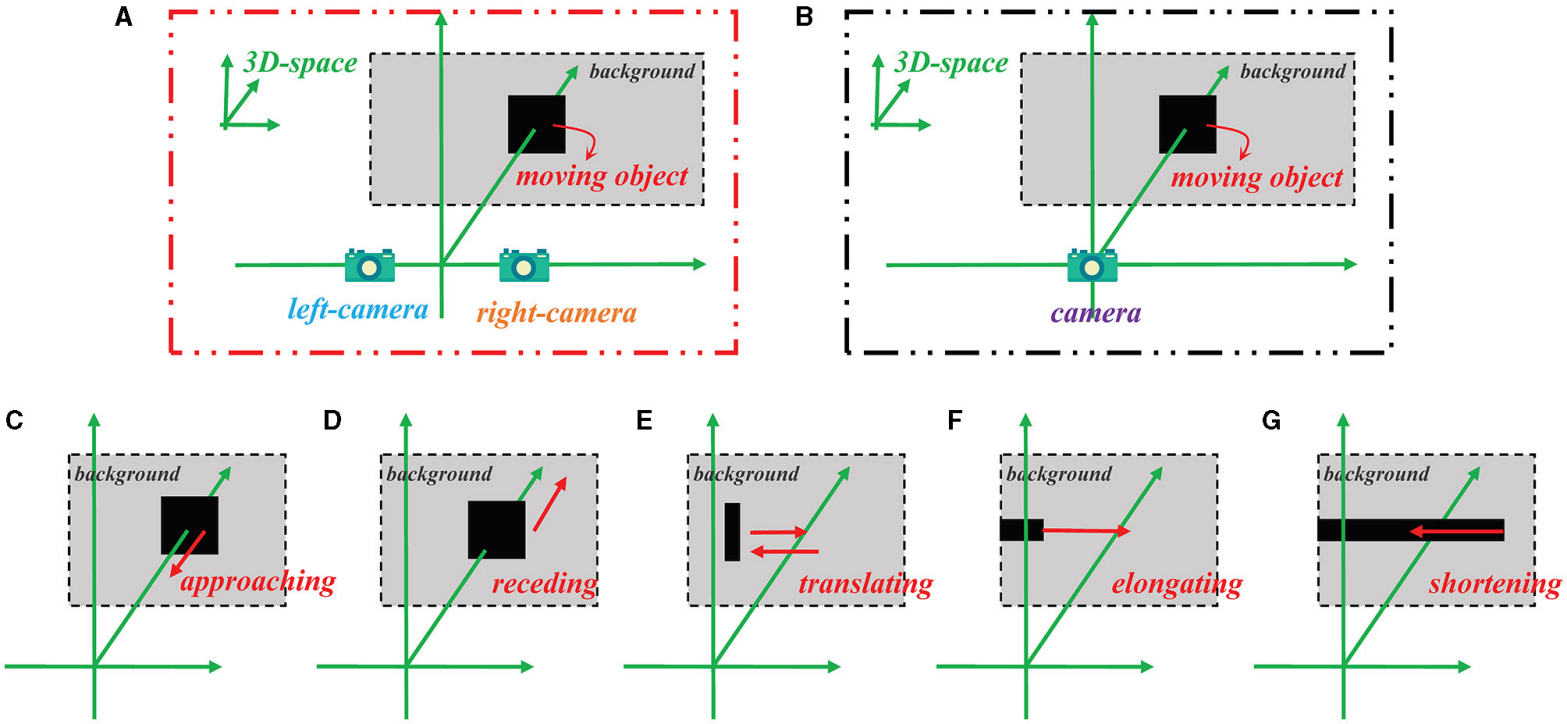
Schematic of how the stimulus videos are generated for the Bi-LGMD model and the comparative model, as well as five specific motion modes. **(A)** Binocular vision input for the Bi-LGMD model. **(B)** Monocular visual input for the comparative model. **(C)** Approaching motion. **(D)** Receding motion. **(E)** Translating motion. **(F)** Elongating motion. **(G)** Shortening motion.

For basic synthetic stimuli testing and model performance testing, all the input visual stimuli are generated using Matlab R2021b according to the projection principle (see Figure 4). The background is set to a solid color, and the pixel has a grayscale value of 0.5. For each frame, the image resolution is 600 × 600 pixels. As to real physical stimuli testing, the input visual stimuli are partly from our own recorded video (rolling ball) and partly from the publicly available KITTI dataset (vehicle scene; Geiger et al., 2013). The image resolutions are 1,280 × 720 pixels and 1,242 × 375 pixels for the videos of the rolling ball and vehicle scene, separately.

All videos are at 30 Hz, and the whole parameters are set according to this sampling rate in the experiments. We list the parameters of the proposed Bi-LGMD model in Table 1. Without special explanation, *n*_*p*_ is 1 and *C*_*T*_ is 15. For the comparative model, the parameters recommended in their literature are used. The computer is equipped with a Core i5 processor with a clock speed of 3.10 GHz, 16 GB of memory, and the operating system is Windows 10. All the experiments are conducted using MATLAB R2021b. The example video clips are shown with results in the following section.

**Table 1:** Setting parameters of the proposed Bi-LGMD model.

**Parameter**	**Description**	**Value**
*n*_*p*_	Luminance change persistence in Equation (1)	0–2
*W*_*I*_	Inhibition weight in Equation (5)	0.3
*C*_*w*_	Constant to calculate *w* in Equation (10)	4
Δ_*c*_	Small real number in Equation (10)	0.01
*T*_*de*_	Decay threshold in *G* layer in Equation (11)	30
*C*_*T*_	Time required to avoid collision in Equation (15)	10–20

### 4.2. Basic synthetic stimuli testing

To verify the basic validity of the proposed Bi-LGMD model, the computer-simulated stimuli are first used for testing. Common basic motion modes include the following five types: approaching, receding, translating, elongating, and shortening. In this section, all the above five types of simulated stimuli are used in the experiments. In addition, grating motion is also chosen for testing as a special phenomenon. As a collision prediction model, the most desirable result would undoubtedly be to respond only to the approaching motion, and not to any other form of movement.

Figure 4 illustrates the method of generating simulated stimulus videos required for the experiments in this section. For the proposed Bi-LGMD model, two cameras are needed to generate video data (see Figure 4A), whereas for the comparative model, only one camera is needed to generate a single video data (see Figure 4B). In addition, Figures 4C–G represent the five basic motion modes mentioned above. These data are generated by Matlab R2021b, simulated by projection transformation of the depth distance and position of the moving object. Moreover, in these experiments, the objects are all moving at a constant speed.

**Figure 5:**
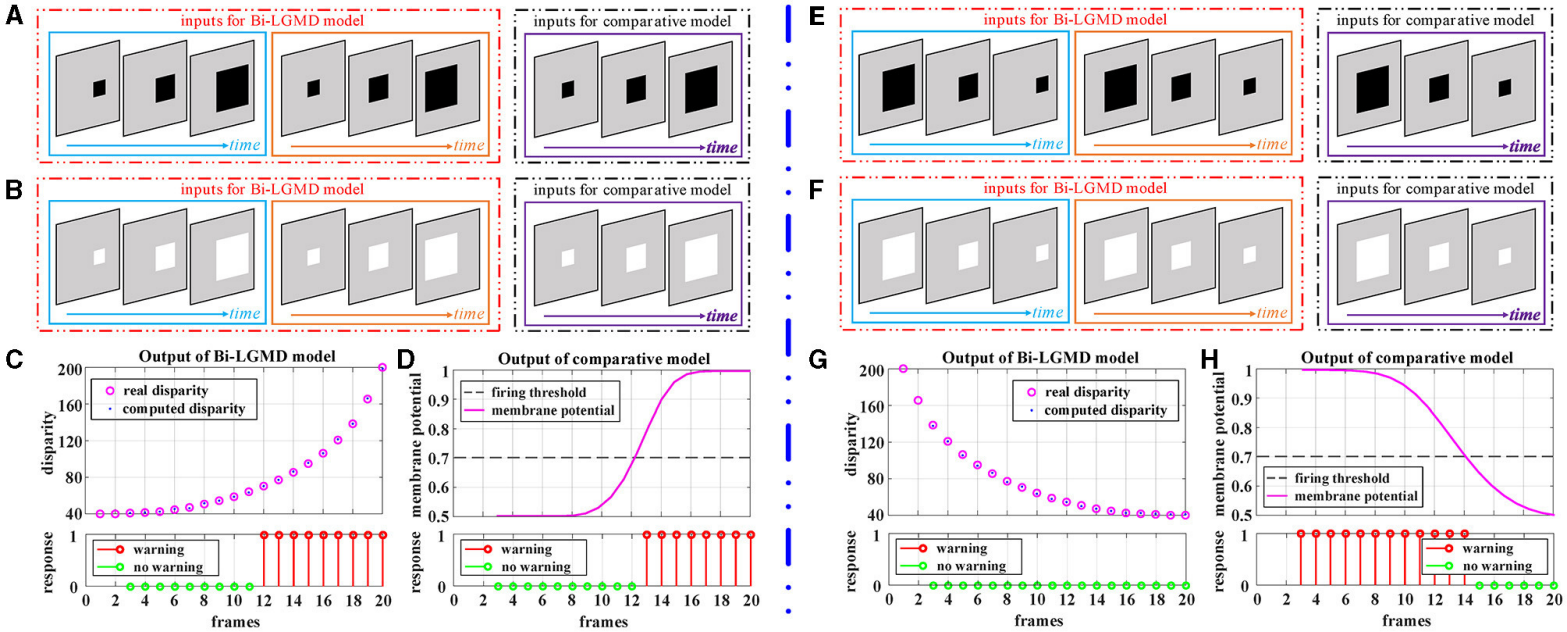
Experimental results of the proposed Bi-LGMD model and the comparative model for simulated stimuli moving in the direction of depth. **(A)** Schematic diagram of an approaching darker object. **(B)** Schematic diagram of an approaching brighter object, with the same motion process as in **(A)**. Identical experimental results for the two sets of simulated stimuli are shown in **(C, D)**. **(C)** The output of the proposed Bi-LGMD model, including the computed disparity (compared with the ground-truth), as well as the final response. **(D)** The output of the comparative model, including the sigmoid membrane potential (SMP), and its comparison to a given hard threshold (set to 0.7). Similarly, for the receding motion in the depth direction, the corresponding schematic and experimental results are presented in **(E–H)** in the same way. Specifically, **(E)** schematic diagram of a receding darker object. **(F)** Schematic diagram of a receding brighter object, with the same motion process as in **(E)**. **(G)** The output of the proposed Bi-LGMD model. **(H)** The output of the comparative model.

Figure 5 corresponds to the situation of two basic motion modes in the depth direction: approaching and receding, where Figures 5A–D show the experimental results of the proposed model and comparative model for the approaching motion, and Figures 5E–H show the experimental results of the two models for the receding motion. For each motion mode, experiments are conducted with darker and lighter objects separately to eliminate the effect of the brightness of the object relative to the background on the experimental results. However, the experimental results show that the brightness of the object has no effect on the results for either the proposed Bi-LGMD model or the comparative model, and a uniform output is given here, as shown in Figures 5C, D, G, H. For the proposed Bi-LGMD model, the computed disparity results are presented in particular, while the ground truth is also marked for comparison. Based on this, the Bi-LGMD model can calculate the depth distance information and obtain a final 0–1 binarized response output. For the comparative model, the sigmoid membrane potential (SMP) is shown and combined with a given hard threshold (set to 0.7), and the same form of response output is obtained for inter-model comparison.

From the experimental results, it can be seen that the disparity calculated by the Bi-LGMD model matches the actual value perfectly. Moreover, the model responds to the approaching stimulus, while it remains unresponsive to the receding process of the object. In fact, as we know, when the object recedes, the disparity of the moving object decreases gradually. Therefore, the model calculates that the depth distance of the object is getting larger, and then, the output of the LGMD layer will be 0, which makes the final result unresponsive. On the contrary, when the object is approaching, in the initial stage, the model calculates that the moving object is far away, so there is no response temporarily. However, as the object gets closer and closer, once the warning depth-distance is reached, the model quickly produces a lasting response.

**Figure 6:**
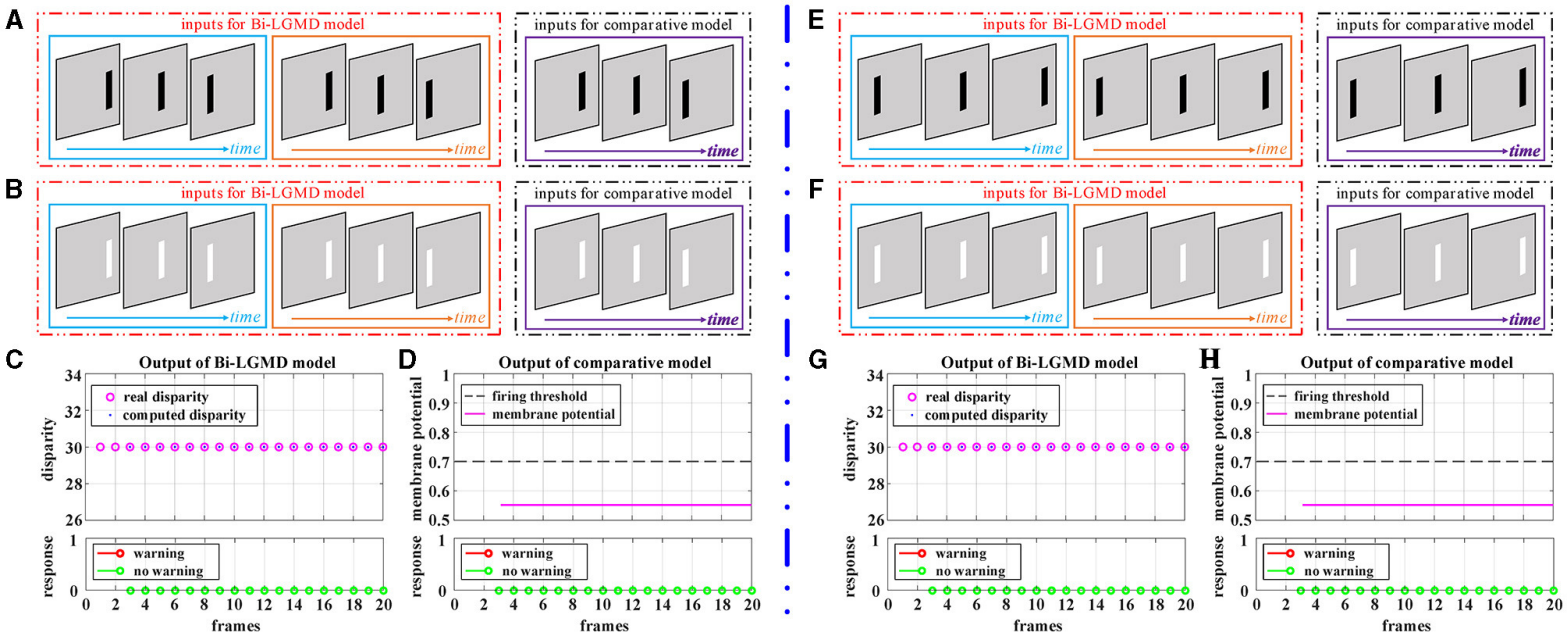
Experimental results of the proposed Bi-LGMD model and the comparative model for simulated stimuli of translating motion (no change in depth distance). **(A)** The translating leftward darker object. **(B)** The translating leftward brighter object. **(C)** The output of the proposed Bi-LGMD model. **(D)** The output of the comparative model. **(E)** The translating rightward darker object. **(F)** The translating rightward brighter object. **(G)** The output of the proposed Bi-LGMD model. **(H)** The output of the comparative model.

Figure 6 shows the experimental results of the proposed Bi-LGMD model and the comparative model for simulated stimuli of translating motion. As can be seen, whether the direction of translation is to the left or to the right, and whether the moving object is darker or brighter, the final response is always 0 for the proposed Bi-LGMD model. In fact, in the three-dimensional real world, when a moving object is translating horizontally, it is always at the same depth distance, so the disparity of the moving object keeps unchanged. The proposed Bi-LGMD model attempts to capture exactly this core feature and, from the computational results, the model does indeed accurately extract the correct disparity results and therefore achieves satisfactory results. For the comparative model, the final response is also always 0. However, as we have seen, the result is based on the comparison of the SMP with a given threshold, so there is conceivably the possibility that the model parameters could have a serious effect on the final result. Furthermore, the fact that the comparative model is based on the summation of the pixel values output from the *G* layer means that the SMP is also affected by the translating speed of the moving object. Overall, the proposed model effectively extracts more essential depth-distance information and will therefore behave more robustly.

**Figure 7:**
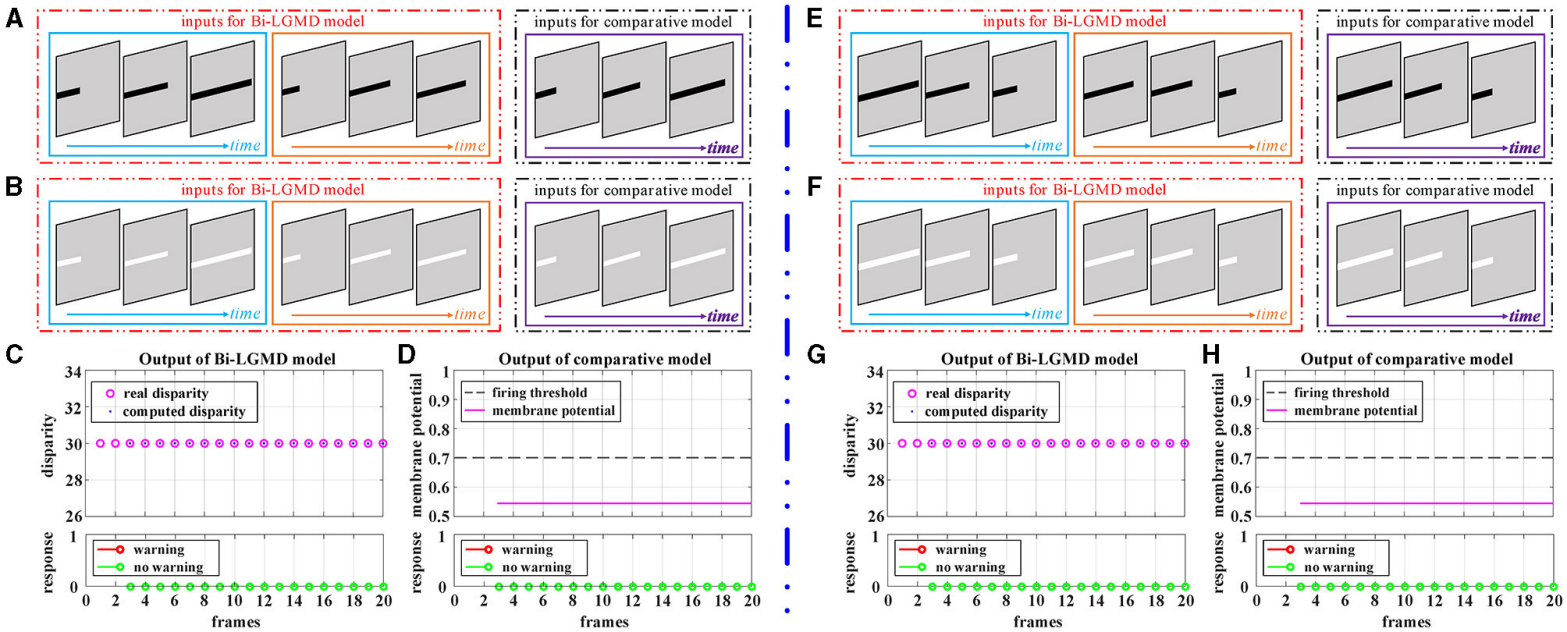
Experimental results of the proposed Bi-LGMD model and the comparative model for simulated stimuli of elongating and shortening motion (no change in depth distance). **(A)** The elongating darker object. **(B)** The elongating brighter object. **(C)** The output of the proposed Bi-LGMD model. **(D)** The output of the comparative model. **(E)** The shortening darker object. **(F)** The shortening brighter object. **(G)** The output of the proposed Bi-LGMD model. **(H)** The output of the comparative model.

Elongating and shortening movements, which are special cases of translating motion, only show a single translating edge due to the limited visual field. However, especially for elongating motion, one-sided changes can easily be confused for edge expansions, which are then misinterpreted by the model as approaching movements. Figure 7 shows the experimental results of the proposed Bi-LGMD model and the comparative model for the moving object in the process of elongating and shortening. As can be seen, the proposed model still extracted the correct disparity information very well and obtained satisfactory experimental results. The experimental analysis for this group of tests is similar to that in translational motion and will not be repeated here.

**Figure 8:**
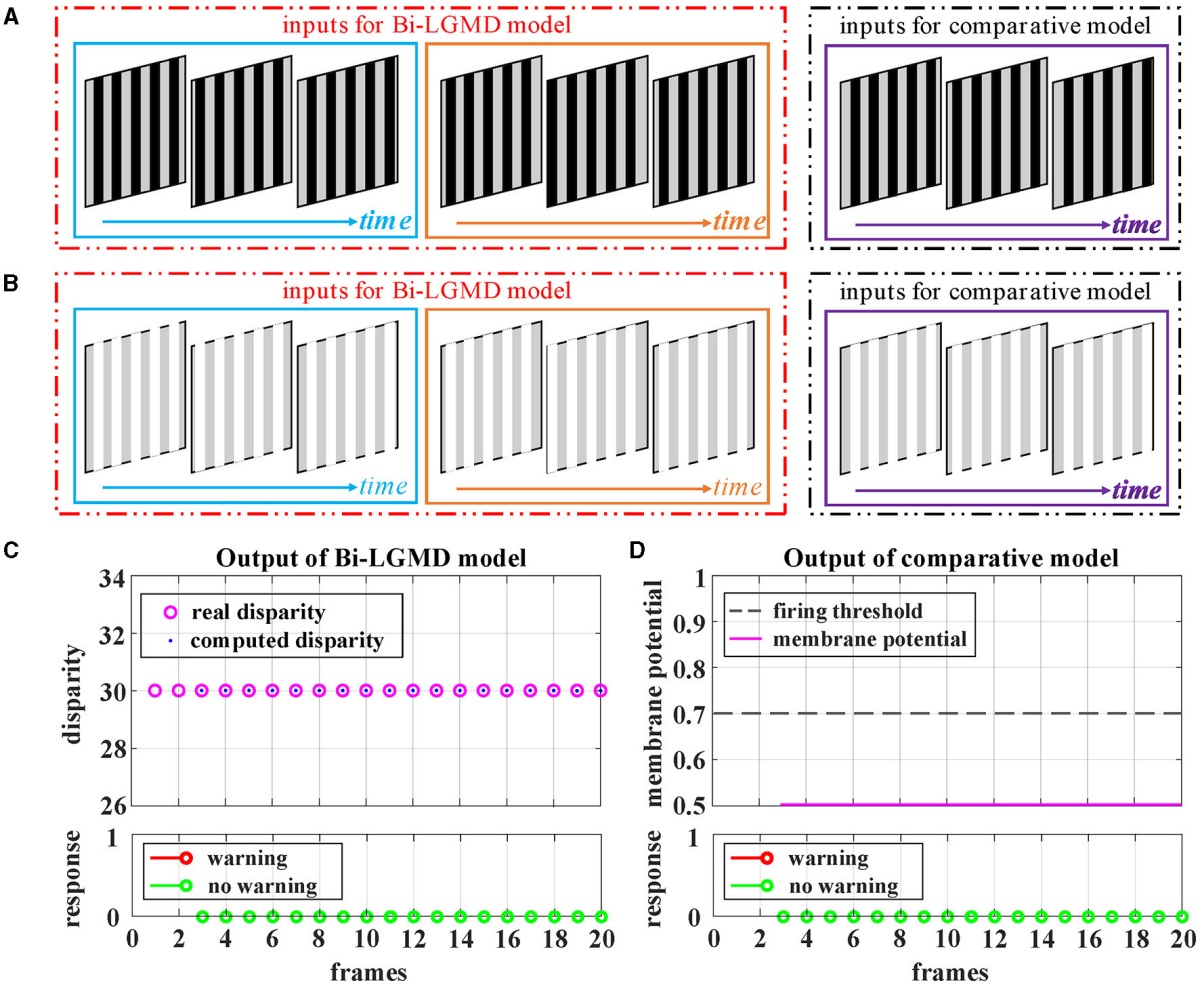
Experimental results of the proposed Bi-LGMD model and the comparative model for simulated stimuli of grating motion (no change in depth distance). **(A)** The grating motion with darker stripes. **(B)** The grating motion with brighter stripes. **(C)** The output of the proposed Bi-LGMD model. **(D)** The output of the comparative model.

Grating movement is a very common phenomenon in our daily life. For example, when the sun shines on the front windshield of a moving car, we can see the bright and dark grating moving stripes from the driver's seat. Obviously, the ideal model does not need to respond to this. However, the grating motion is always accompanied by the luminance change of the whole field, resulting in an easily observable response in the model. In order to suppress this unnecessary response, the existing LGMD-based models introduce the feedforward inhibition (FFI) mechanism. However, no evidence has been found to show how feedforward inhibition could increase the selectivity for approaching over receding objects (Keil and Rodriguez-Vazquez, 2003), and from the perspective of biological neurology, there are still some doubts about the explanation and rationality of it. Moreover, the parameter setting of the FFI mechanism itself is also a relatively complex problem. Figure 8 shows the experimental results of the proposed Bi-LGMD model and the comparative model for simulated stimuli of grating motion. As can be seen, both models achieve the desired non-response result. However, the two models do not work in the same way. The proposed Bi-LGMD model is based on the computed disparity information, and since there is no change in depth distance, it is judged that there is no collision risk. In the comparative model, the FFI mechanism is triggered by the change of pixel gray value in a large area, forcing the response of the model to be suppressed. It is worth noting that the spacing between the grating stripes, and the moving speed, moving direction as well as the brightness of the grating stripes will not affect the experimental results of the proposed Bi-LGMD model. In fact, the “disparity” and “depth distance” are always the essence in any case, and they are not affected by the above factors. Therefore, the Bi-LGMD model can easily judge the grating motion as a translating motion.

So far, in all five basic motion modes as well as the grating motion, the Bi-LGMD model only responds to the approaching motion, while remaining unresponsive to any other motion modes, which fully meet our expectations. Moreover, such response results are independent of the brightness of the moving object. These results are largely due to the fact that the Bi-LGMD model obtains the depth distance of the object by calculating the disparity, and thus further effectively distinguishing the approaching motion mode from others. Actually, according to the computed disparity, the Bi-LGMD model can clearly classify various specific motion modes into the following three categories: approaching, receding, and translating. In addition, for approaching motion, the model will further extract the approaching velocity at each time step, combined with the current depth distance information, the model only generates a collision warning if it actually perceives the threat of an imminent collision, that is, if the object reaches a dynamically adaptive warning depth-distance.

### 4.3. Real physical stimuli testing

In the previous section, the validity and superiority of the proposed Bi-LGMD model is initially verified by simulated stimuli. In this section, real physical stimuli are used for testing. Compared with the computer-simulated stimuli, the biggest difference is that there is more environmental noise in the real physical scenes, such as shadows, reflections, etc. In addition, the motion speed and motion state of moving objects are also relatively unstable. Therefore, visual stimulation in real physical scenes is undoubtedly a more difficult challenge for the collision prediction task, but at the same time, it is also one of the important criteria to evaluate the performance of the model.

**Figure 9:**
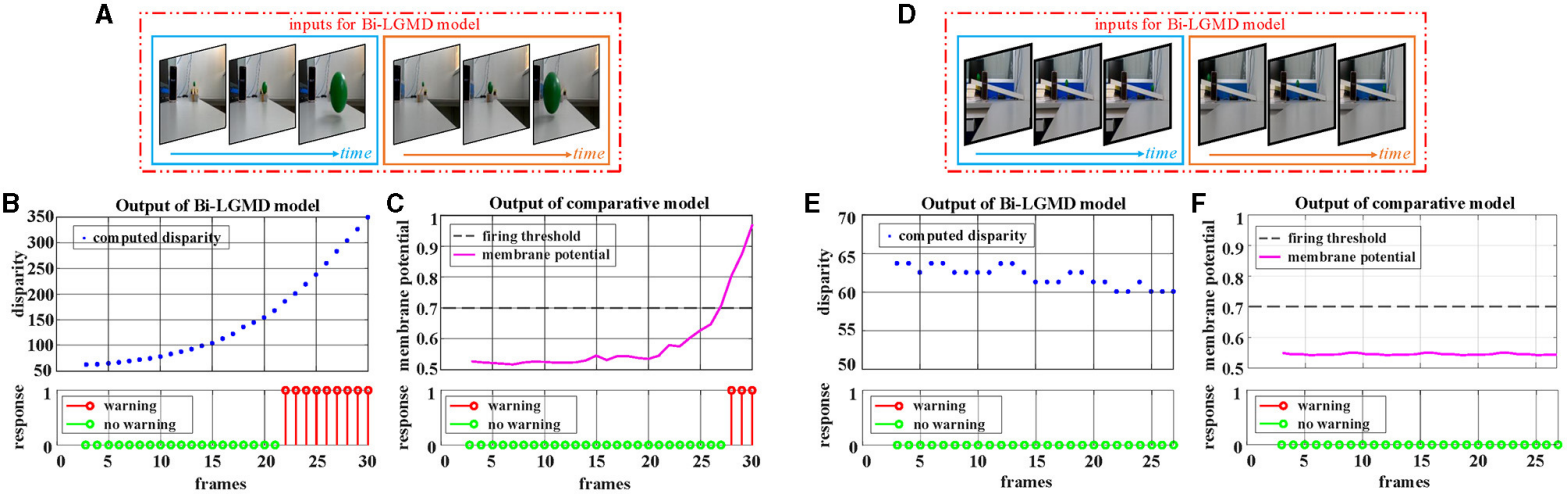
Experimental results of the proposed Bi-LGMD model and the comparative model for real scene videos of indoor moving ball. **(A)** The input image streams of a approaching ball. The blue and orange boxes indicate inputs from the left and right cameras, respectively. **(B)** The output of the proposed Bi-LGMD model, including the computed disparity, as well as the final response. **(C)** The output of the comparative model, including the sigmoid membrane potential (SMP), and its comparison to a given hard threshold (set to 0.7). Similarly, for the translating ball, the corresponding schematic and experimental results are presented in **(D–F)** in the same way. Specifically, **(D)** the input image streams of a translating ball. **(E)** The output of the proposed Bi-LGMD model. **(F)** The output of the comparative model.

Firstly, the videos of a small moving ball taken indoors are used for testing. Two GoPro motion cameras of the same model (Hero 8 Black) are used to capture the scene simultaneously. The optical axes are kept parallel throughout the entire shooting process. The experimental results are shown in Figure 9. In the approaching ball video, the green ball is approaching from a distance along a fixed oblique track. Due to a certain inclination of the track, under the action of gravitational potential energy, the approaching speed of the ball gradually accelerates, and the ball bounces on the table after it gets off the track in the later stage. There are obvious shadows, reflections, and so on in the video. It can be seen from the experimental results that both models produce an early warning response, in which the proposed model has an earlier warning time, while the comparative model produces the early warning response in the late stage when the ball's approaching speed is faster. Reverse the video sequence to simulate the receding process, and the Bi-LGMD model has no response to that because the computed disparity is getting smaller over time. However, the comparative model has two early warnings at the beginning. The outputs are not shown here for brevity. For the translating ball video (in fact, it is difficult to ensure that the ball moves strictly in translation, so the ball is not always at the same depth distance. The so-called translation here is just a rough visual effect.), no warning response was generated for both models.

**Figure 10:**
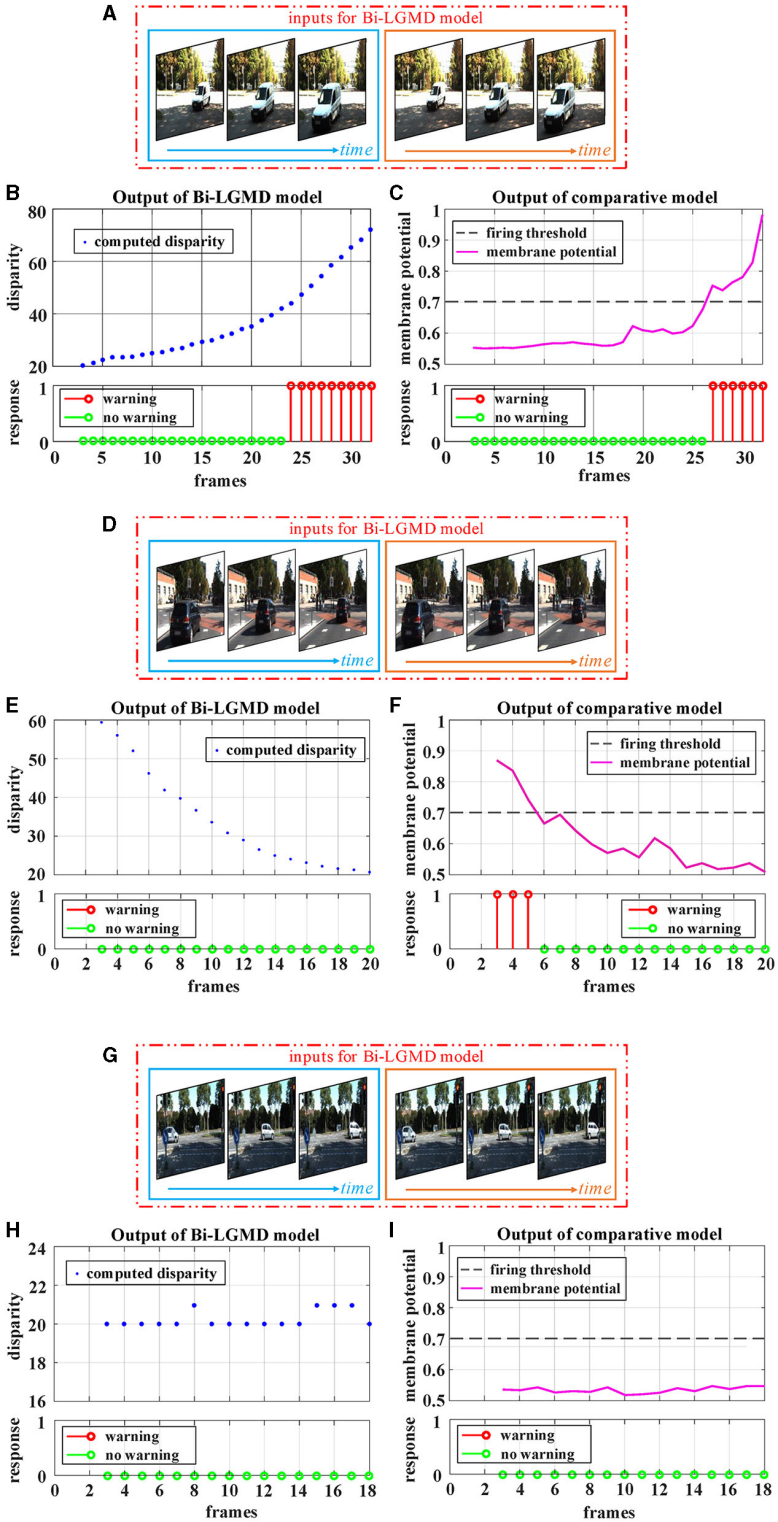
Experimental results of the proposed Bi-LGMD model and the comparative model for real scene videos of outdoor moving vehicle. There are three sets of experiments, each showing examples of the input image streams and the corresponding output of the two models. **(A)** The input of a approaching vehicle. **(D)** The input of a receding vehicle. **(G)** The input of a translating vehicle. The blue and orange boxes indicate inputs from the left and right cameras, respectively. **(B, E, H)** Are the outputs of the proposed Bi-LGMD model, including the computed disparity, as well as the final responses. **(C, F, I)** Are the outputs of the comparative model, including the sigmoid membrane potential (SMP), and its comparison to a given hard threshold (set to 0.7).

Following that, the outdoor vehicle scene videos are used for testing. Here, the public KITTI data set is adopted. For each of the three basic motion modes, a video is chosen for the experiment, as shown in Figure 10. For approaching motion, a white vehicle is approaching from the front as shown in Figure 10A. For receding motion, a black vehicle drives away as shown in Figure 10D. For translating motion, a white car moves from the left to the right in the field of view as shown in Figure 10G. It can be seen that the experimental results of the proposed Bi-LGMD model are fully in line with expectations, and can effectively calculate parallax and obtain satisfactory model output based on depth and distance information. Compared with the comparative model, the explainability and robustness of the Bi-LGMD model are stronger, especially for the backward motion, the Bi-LGMD model shows better experimental results.

### 4.4. Model performance testing

As a binocular LGMD-based visual neural network for collision prediction, Bi-LGMD is fundamentally different from the existing models in many aspects. The estimation of the depth distance of a moving object, indeed, brings great benefits to the model. In this section, we will discuss this in detail, and analyze the advantages of Bi-LGMD by comparing it with existing models. In the following experimental comparison, since monocular and binocular stimuli need to be generated correspondingly, we use computer-simulated synthetic stimuli to carry out the experiment.

#### 4.4.1. Sensitivity to model parameters

Parameters are undoubtedly crucial for any model and even have a direct impact on the model results. In this part, the topic of parameters of Bi-LGMD and existing models will be discussed. In fact, as we can see, the basic process (*P*, *E*, *I*, *S*, *G* layers) of the proposed Bi-LGMD model is consistent with existing models, therefore the parameters after the *G* layer will mainly be discussed.

In existing models, the following function is used to activate the summation result of *G* layer as the output of the LGMD layer (representing the membrane potential of the LGMD neuron). After that, a given firing threshold *T*_*fir*_ is used to determine whether the LGMD neuron is activated, such as Yue and Rind (2006), Fu et al. (2018b, 2019b, 2020), Luan et al. (2021), Lei et al. (2022), and Li et al. (2022).


(17) $$LGMD(t)={\left(1+exp\left(\frac{-\sum_{x=1}^{R}\sum_{y=1}^{C}\hat{G}(x,y,t)}{\alpha \cdot R\cdot C}\right)\right)}^{-1}$$

Therefore, there are two important parameters involved: α and *T*_*fir*_. Obviously, the existing models must fully consider the problem that the given threshold should roughly match the activation result, which is actually relatively difficult to adjust adaptively. As we know, the sigmoid function curve *y*(*x*) = [1+*exp*(−*x*)]^−1^ increases monotonically, with a range of 0.5–1. For a standard collision process that gradually approaches from a distance, the ideal sigmoid activation result should be approximately from 0.5 to nearly 1, which requires that the parameter α is very suitable so that the ratio $\overset{R}{\underset{x=1}{\sum }}\overset{C}{\underset{y=1}{\sum }}\hat{G}\left(x,y,t\right)/\left(\alpha \cdot R\cdot C\right)$ could almost fill the interval [0, 3] since *y*(3)≈0.9526. In other words, if the α is chosen too large so that the ratio is very small, the sigmoid activation results will be all near 0. Conversely, if the α is chosen too small, resulting in the ratio being basically >3, the sigmoid activation results will be all around 1. Obviously, in these cases, it is difficult to match the sigmoid activation results with the given thresholds. In addition, it can be seen from the formula that the value of α will also be affected by the image sizes *R* and *C*, which means that for the same collision scenario, cameras with different resolutions or different fields of view will have a serious impact on the model, which makes it more difficult to determine the parameters α. In summary, the existing models are very sensitive to the above two parameters (α and *T*_*fir*_), making them less robust.

By contrast, in the Bi-LGMD model, there is only one parameter *D*_*W*_ after *G* layer. Furthermore, this parameter *D*_*W*_(*t*) is adaptively adjusted with the motion state of the object at each time step. In more detail, *D*_*W*_ is linearly determined by *C*_*T*_ for convenience in our case, and *C*_*T*_ is given a very clear realistic physical meaning, which can be used as a guide for adjusting.

**Figure 11:**
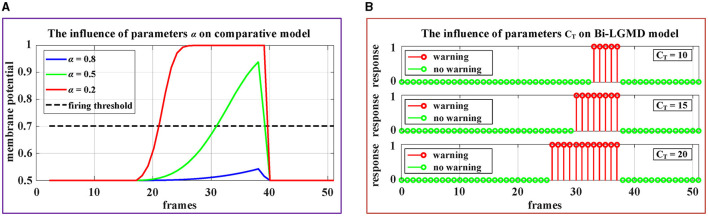
Experimental results of the effects of parameters on the comparative model and the proposed Bi-LGMD model. **(A)** The experimental results of the comparative model. **(B)** The experimental results of proposed Bi-LGMD model.

In addition, Figure 11 demonstrates the impact of these parameters on the proposed model and the comparative model. The video stimuli used in the experiment were simulated approaching black blocks similar to those shown in Figure 4C. To more comprehensively illustrate the impact of parameters on the model, we set the following motion pattern: the object remains stationary for the first 15 frames, then begins to approach and stops approaching at frame 37. The speed remains constant during the approaching process.

It can be seen from the experimental results that the parameter α has a great impact on the sigmoid membrane potential results of the existing models, and if an inappropriate value α is selected, the existing models will fail (under the given firing threshold *T*_*fir*_). Contrastingly, the influence of parameter *C*_*T*_ on the results of the proposed Bi-LGMD model is mainly reflected in the early warning response time. Specifically, the larger the *C*_*T*_ value, the earlier the early warning response time. However, the Bi-LGMD model will always produce a warning before the collision. In addition, according to the actual physical meaning of *C*_*T*_, we can reasonably adjust the value range of *C*_*T*_ based on the system performance.

Hence, the proposed model has fewer parameters and is more robust than the existing model. In terms of parameter adjustment, the proposed model has more clear guiding significance, so it can be considered that the proposed model is superior to the existing models in this respect.

#### 4.4.2. Adaptability to motion modes

**Figure 12:**
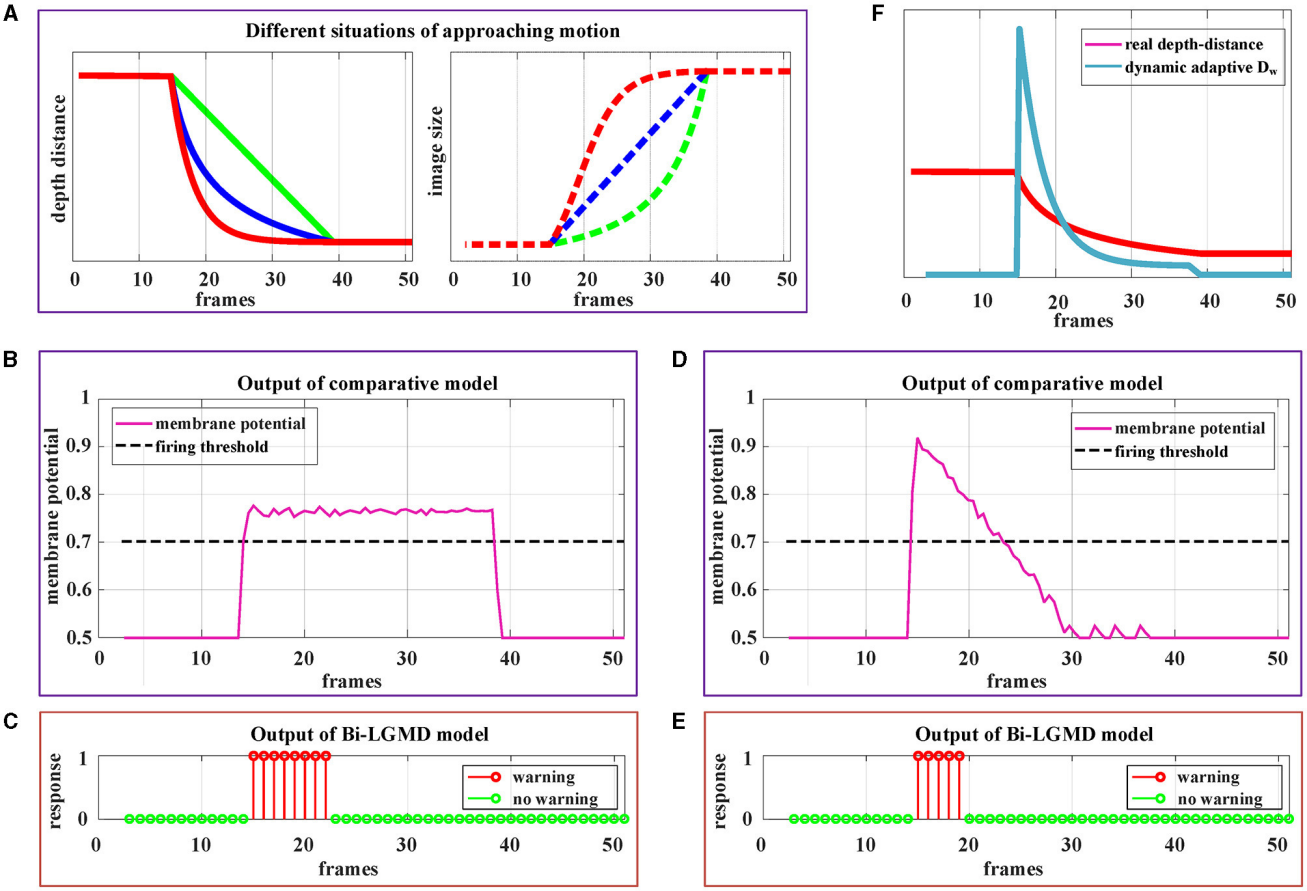
Experimental results of the proposed Bi-LGMD model and comparative model for three different approaching modes. **(A)** The specific details of three different approaches modes. Mode represented by the green line is approaching at a constant speed. Mode represented by the blue line is a special deceleration approach, leading to a linear increase in imaging size. Mode represented by the red line is also a deceleration approach, leading to a gradual decrease in the increment of imaging size. **(B)** The output of the comparative model for the approaching mode represented by the red blue in **(A)**. **(C)** The output of the proposed Bi-LGMD model for the approaching mode represented by the blue line in **(A)**. **(D)** The output of the comparative model for the approaching mode represented by the red line in **(A)**. **(E)** The output of the proposed Bi-LGMD model for the approaching mode represented by the red line in **(A)**. **(F)** The dynamic adaptive warning depth distance (*D*_*W*_) and depth distance in **(E)**.

By estimating the depth distance of a moving object at each time step, the Bi-LGMD model accurately identifies its motion modes, as seen in the previous experiment. To further fully illustrate the advantage of estimating depth distance, more detailed motion patterns are used for testing. Since the Bi-LGMD model does not respond to receding and translating motion, we mainly take the approaching motion as an example to illustrate. In particular, unlike the previous experiments in which the object is always moving at a constant velocity, we will explore other different approaching cases. Similar to the experimental setup in Figure 11, during the first 15 frames and the last 15 frames, the object remains stationary in the simulated stimulus video. The experimental description and results are shown in Figure 12.

Figure 12A depicts three different approaching patterns in terms of depth distance and image size over time, represented by different colors. Among them, the mode represented by the green line is approaching at a constant speed (Marked as **Approaching Pattern 1**), which is the pattern set in all previous experiments. In particular, the pattern represented by the blue line is a special deceleration approach, leading to a linear increase in imaging size (Marked as **Approaching Pattern 2**). The pattern represented by the red line is also a deceleration approach, leading to a gradual decrease in the increment of imaging size (Marked as **Approaching Pattern 3**).

Figures 12B, C shows the experimental results of the comparative model and the proposed Bi-LGMD model for the Approaching Pattern 2. As can be seen, the SMP output of the comparative model is almost a horizontal straight line, indicating that the activity of LGMD cells is always maintained at the same level. In fact, parameter α does not change the overall shape of the response, so that the model either reaches the given firing threshold at the beginning of movement or never, both of which are not the ideal results. Such experimental results are directly related to the fact that the imaging size varies linearly. By contrast, the Bi-LGMD model only outputs 1 for the first few frames when the object begins to approach, and 0 for the rest of the time. This result is actually reasonable. As can be seen, the approaching speed is very fast at the beginning, so the model needs to trigger an early warning immediately. However, when the approaching speed of the moving object gradually slows down, there is no collision threat temporarily, so the output changes to 0. The warning depth-distance *D*_*W*_ of each time step obtained from the model is shown in **(E)**, when the approaching speed slows down, the warning depth-distance *D*_*W*_ decreases accordingly, which reflects its dynamic adaptive process. Similarly, Figures 12D, E shows the experimental results of the comparative model and the proposed Bi-LGMD model for the Approaching Pattern 3.

In summary, the comparative model is not well-adapted to various approach models, while the Bi-LGMD model can achieve satisfactory results based on depth distance estimation, as well as the dynamic adaptive warning depth distance mechanism.

#### 4.4.3. Robustness to the input image streams

Robustness is one of the important indexes for model evaluation. In the existing models, the quality of the input image streams has a certain impact on the results, which makes the model not robust enough. In this section, we select two key factors affecting image quality (contrast and noise) for testing. We make a detailed analysis based on the results, and further compare the differences between Bi-LGMD and the existing models.

##### 4.4.3.1. Contrast

The contrast between the moving object and the background is obviously a very important factor. In this group of experiments, since both the background and the moving object are set to a solid color, the contrast ratio can be simply regarded as the gray value of the background (the gray value of the moving darker object is set to 0). Intuitively, the greater the contrast, the easier it is for the model to recognize moving objects and successfully perceive collisions. But as the contrast gradually decreases, the task of sensing collisions becomes more difficult.

**Figure 13:**
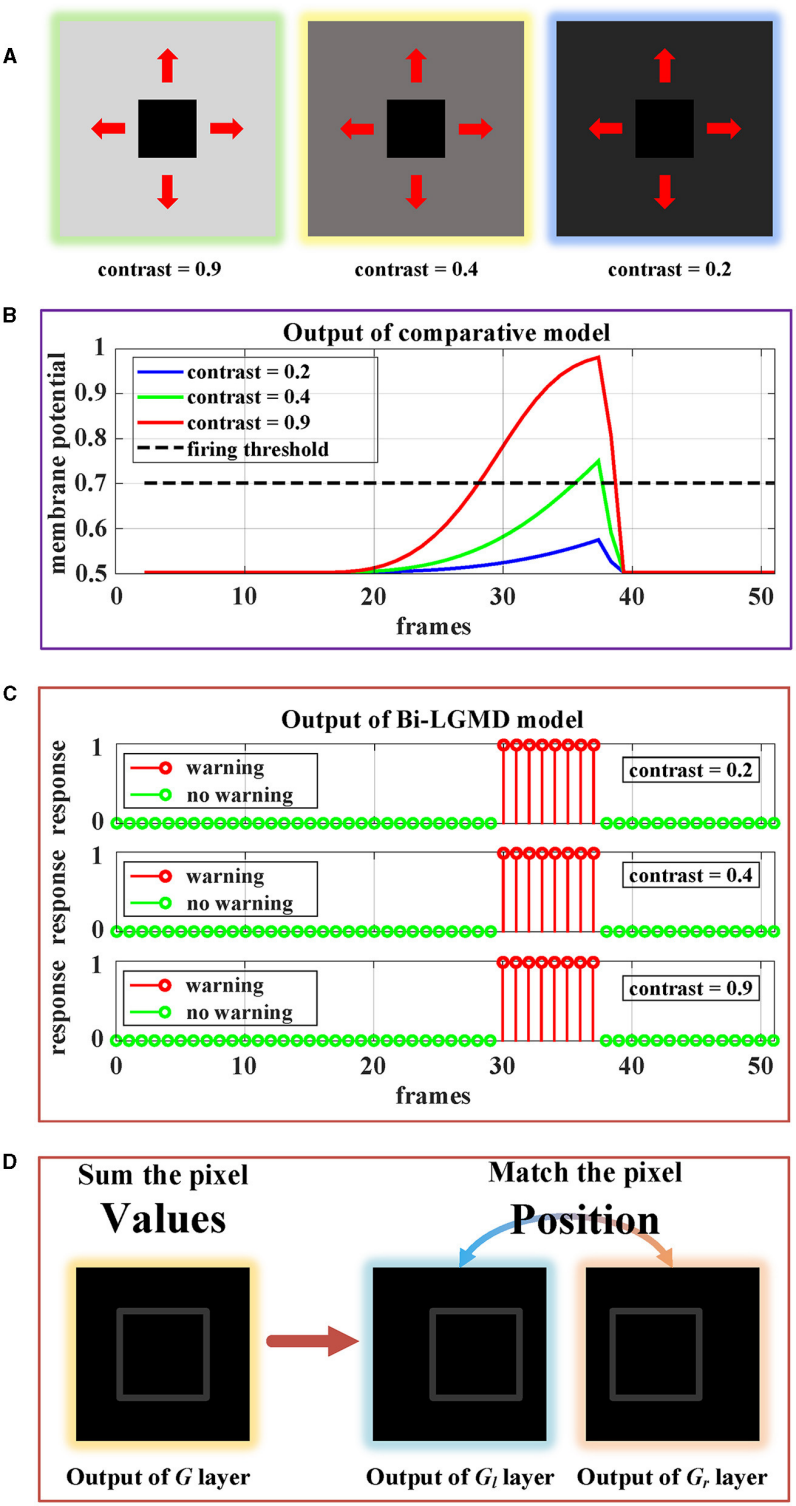
Experimental results of the proposed Bi-LGMD model and the comparative model for same approaching process with different contrast. **(A)** Visual examples in three different contrasts, decreasing from left to right. **(B)** Experimental results of the comparative model. **(C)** Experimental results of the proposed Bi-LGMD model. **(D)** Schematic of the essential differences between the two models when dealing with low contrast problems.

Figure 13 shows the approaching motion with three different contrasts. The motion process is based on the Approaching Pattern 1 shown in Figure 12A. For the above three cases, we generated monocular data and binocular data according to the imaging principle. As can be seen, in the comparative model, the higher the contrast, the stronger the activation result of the sigmoid membrane potential of the LGMD cell. Therefore, in the case of low contrast, the activation result is far lower than the given threshold, which makes the model unable to successfully perceive collisions and generate early warnings. However, for the proposed Bi-LGMD model, even if the contrast is small enough, the output of the model is still not affected at all. It is because the Bi-LGMD model does not care about the pixel value, but only needs to match the relevant position of the moving object from the left and right camera to obtain the correct disparity, so as to determine the depth distance of the moving object. As shown in Figure 13D, the model converts the focus from pixel value to corresponding position matching, which is a major difference in the Bi-LGMD model. Under this change of thinking, the model does not rely on the absolute size of the pixel value, so no matter how the contrast is, the pixel position matching is still accurate. Therefore, the contrast factor has no effect on the estimation of the depth distance of the moving object, so naturally, it does not affect the final effect at all.

##### 4.4.3.2. Image noise

In the previous section, all synthetic stimuli used in the experiment are clean. However, the input image streams in the real world are always accompanied by different kinds and degrees of noise, which is caused by hardware equipment and other factors. In other words, noise is often an inevitable objective factor in image sampling. To test the robustness of the model to noise, different levels of White Gaussian Noise are randomly added to the synthetic stimulus.

**Figure 14:**
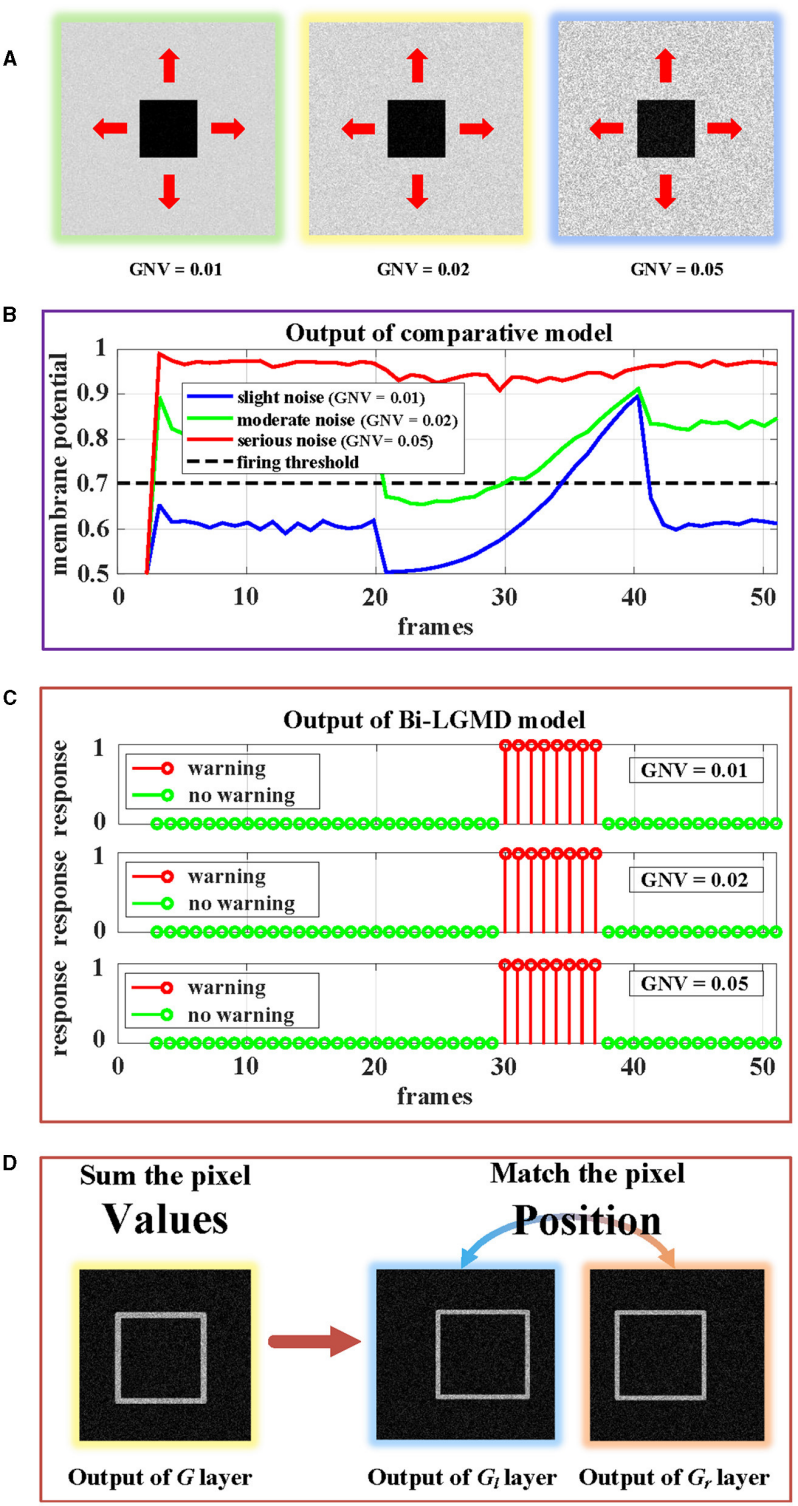
Experimental results of the proposed Bi-LGMD model and the comparative model for same approaching process with different levels of noise. **(A)** Visual examples in three different levels of noise, increasing from left to right. **(B)** Experimental results of the comparative model. **(C)** Experimental results of the proposed Bi-LGMD model. **(D)** Schematic of the essential differences between the two models when dealing with noise.

Similar to the experiment on contrast, there are three groups of approaching processes with different levels of noise, as shown in Figure 14. Gaussian noise variances (GNV) from left to right are 0.01 (slight noise, green), 0.02 (moderate noise, yellow), and 0.05 (serious noise, blue), respectively. It can be seen that noise has a serious impact on existing models, while the Bi-LGMD model is very robust. The reasons here are the same as those mentioned above. For the existing model, the noise seriously affected the pixel value, thereby affecting the results of the model. However, for Bi-LGMD, the matching of corresponding positions is relatively stable.

## 5. Further discussion

As a research based on binocular LGMD visual neural network, this paper proposes a novel model with depth distance as the essential feature, and verifies the feasibility and superiority of this idea through systematic experiments. In fact, the advantages of introducing depth distance into models are not limited to the work described in this paper. On the basis of the proposed Bi-LGMD model, there are more research directions worth exploring in the future.

Two points are briefly listed here: (1) For the case of multiple moving objects, the existing LGMD-based models are difficult to obtain ideal results due to the mixture of multiple stimuli. However, based on the proposed Bi-LGMD model, it is possible to distinguish moving objects at different depth distances and obtain the motion pattern of each object to achieve better model results. (2) More exploration of the approaching azimuth of the moving object can be attempted. Obviously, as a collision prediction model, it needs to respond strongly to stimuli that approach directly from the front, while it does not need to respond to the oblique approach motion such as passing-by. Based on the Bi-LGMD model and making full use of depth distance information, these ideas above will be our follow-up research.

## 6. Conclusion

This paper presents a LGMD-based neural network with binocular vision for collision prediction. In this model, the depth-distance information of moving objects is further taken into account, which enables the model to correctly distinguish between approaching and other modes of motion, and the model results are more interpretable. Moreover, the early warning depth-distance parameter in the proposed model is designed to be dynamically adaptive, which allows the model to generate early warnings at the most appropriate time depending on the individual performance of the system, which is a great improvement over existing LGMD-based models. The model no longer depends on the activation function and a given hard threshold, which mitigates the sensitivity to model parameters. The proposed Bi-LGMD visual neural network model is systematically tested on synthetic stimuli and real-world scene videos, showing that it is effective and robust to input quality, such as noise, low contrast, and other factors. Unlike existing LGMD-based models that rely heavily on image pixel values, the Bi-LGMD model shifts the focus to position matching, which may be a new line of research to consider in the future.

## Data availability statement

The raw data supporting the conclusions of this article will be made available by the authors, without undue reservation.

## Author contributions

YZ, HL, and JP contributed to conception and design of the study. YZ and YW organized the database. JP performed the statistical analysis. YZ wrote the first draft of the manuscript. YZ, YW, and GW wrote sections of the manuscript. All authors contributed to manuscript revision, read, and approved the submitted version.
